# Transcriptomic and Proteomic Landscape of Sugarcane Response to Biotic and Abiotic Stressors

**DOI:** 10.3390/ijms24108913

**Published:** 2023-05-17

**Authors:** Ao-Mei Li, Fen Liao, Miao Wang, Zhong-Liang Chen, Cui-Xian Qin, Ruo-Qi Huang, Krishan K. Verma, Yang-Rui Li, You-Xiong Que, You-Qiang Pan, Dong-Liang Huang

**Affiliations:** 1 Key Laboratory of Sugarcane Biotechnology and Genetic Improvement (Guangxi), Ministry of Agriculture and Rural Affairs/Guangxi Key Laboratory of Sugarcane Genetic Improvement/Sugarcane Research Institute, Guangxi Academy of Agricultural Sciences, Nanning 530007, Chinaliyr@gxaas.net (Y.-R.L.); 2 Key Laboratory of Sugarcane Biology and Genetic Breeding, Ministry of Agriculture and Rural Affairs/Key Laboratory of Ministry of Education for Genetics, Breeding and Multiple Utilization of Crops, College of Agriculture, Fujian Agriculture and Forestry University, Fuzhou 350002, China; queyouxiong@fafu.edu.cn

**Keywords:** sugarcane, transcriptome, proteome, gene, miRNA, stress

## Abstract

Sugarcane, a C_4_ plant, provides most of the world’s sugar, and a substantial amount of renewable bioenergy, due to its unique sugar-accumulating and feedstock properties. Brazil, India, China, and Thailand are the four largest sugarcane producers worldwide, and the crop has the potential to be grown in arid and semi-arid regions if its stress tolerance can be improved. Modern sugarcane cultivars which exhibit a greater extent of polyploidy and agronomically important traits, such as high sugar concentration, biomass production, and stress tolerance, are regulated by complex mechanisms. Molecular techniques have revolutionized our understanding of the interactions between genes, proteins, and metabolites, and have aided in the identification of the key regulators of diverse traits. This review discusses various molecular techniques for dissecting the mechanisms underlying the sugarcane response to biotic and abiotic stresses. The comprehensive characterization of sugarcane’s response to various stresses will provide targets and resources for sugarcane crop improvement.

## 1. Introduction

In this dynamic era of climate change, growing crops under stressful environmental conditions is inevitable for many farmers. However, it also contributes to substantial yield reductions on a global scale [1,2,3,4]. Plants have acquired complex and coordinated signaling and response mechanisms to survive exposure to both biotic and abiotic stressors. In particular, cellular-level response mechanisms are crucial for maintaining plant growth, yield, and development in unfavorable conditions [5,6]. In order to maintain regular cellular and metabolic activities, significant changes in gene expression were observed in highly tolerant plants [7]. In plants, biomass accumulation is positively correlated with the differential diurnal biosynthesis of metabolites. Photosynthetic processes produce organic substances used for growth, development, and metabolic activities [8,9].

Sugarcane (hybrids of *Saccharum* spp.), the source of approximately 80% of global sugar production [10], exhibits marked diurnal variation in non-structural carbohydrate production [9]. In addition, approximately 50% of the total sugarcane biomass is used for the production of bioethanol [11,12,13]. Modern sugarcane cultivars are genomically diverse, with a greater extent of polyploidy and aneuploidy, and combine the high sugar accumulation of *S. officinarum* (approximately 20–70% of the genomic contribution) with the ratooning and hardiness of *S. spontaneum* (approximately 10–20% of the genomic contribution) [14,15]. Unfortunately, traditional breeding methods for the improvement of sugarcane stock are challenging due to genomic complexity and a narrow gene pool [16].

**Figure 1:**
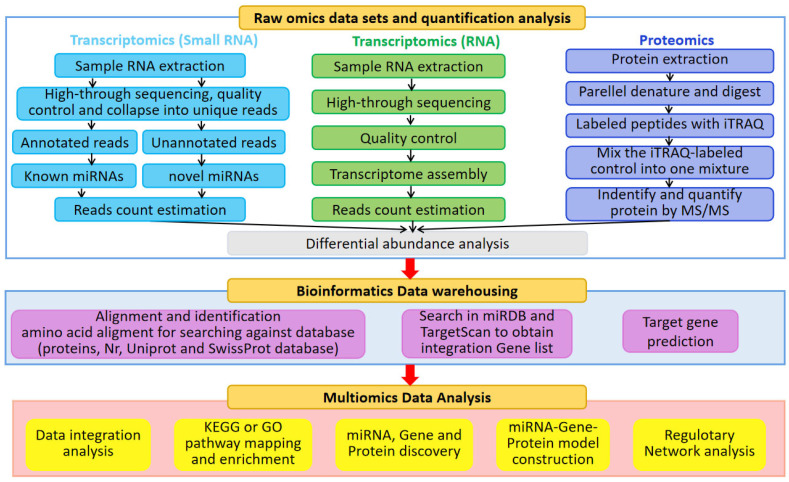
**A generalized transcriptomic and proteomic workflow for sugarcane.** The quantification of transcripts, proteins, and miRNAs, bioinformatics analysis, and multiomics analysis are included.

Recently, transcriptomic and proteomic techniques have been used to study the molecular basis underlying sugar production and stress resistance in sugarcane. The application of these techniques has revealed new information regarding the molecular mechanisms and adaptations responsible for sugarcane sensitivity and tolerance, and has resulted in productive gains [17,18]. Transcriptomics and proteomics are facilitated by the discovery and characterization of the genes, proteins, and regulatory networks underlying desired agronomic traits. Specifically, these approaches rely on sequencing, bioinformatics, and computational analysis. Figure 1 shows a generalized workflow for sugarcane omics. The precise quantification of transcripts and proteins is fundamental for differential abundance analyses. Based upon these analyses, bioinformatics and multiomics approaches can provide novel insights into complex phenomena.

**Figure 2:**
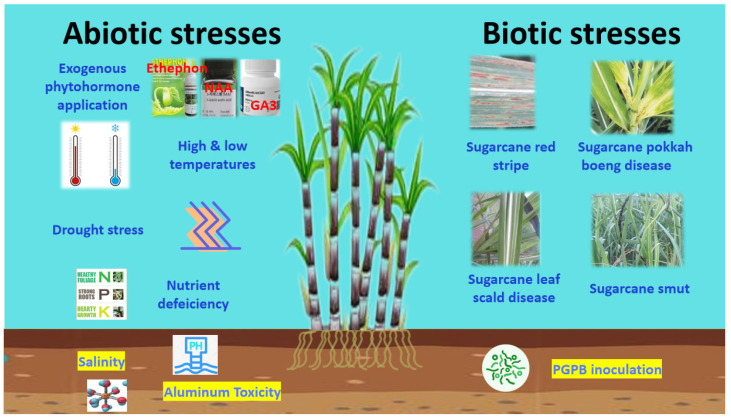
Abiotic and biotic stresses encountered by sugarcane.

A multitude of stressful conditions are encountered by, and often hamper the growth of, sugarcane (Figure 2) [19,20,21,22]. Extreme temperatures, drought, soil salinity, metal toxicity, and nutrient deficiency are a few of the major abiotic stressors faced during the process of sugarcane growth. The primary diseases affecting sugarcane growth and yield include, but are not limited to, red stripe [23], pokkah boeng [24], leaf scald [25], and smut [26]. Exploring how gene expression is reprogrammed in sugarcane will be crucial for understanding how this important crop responds to stressful conditions and biotic challenges, and will open new avenues for crop improvement.

## 2. Sugarcane Transcriptomic Analysis

**Figure 3:**
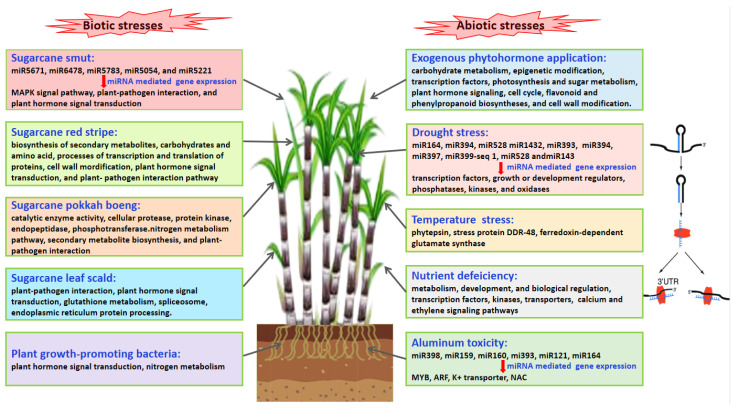
The sugarcane stress response involves multiple biological processes, signal transduction pathways, and miRNA-mediated gene expression regulation.

The transcriptome represents a collection of all the transcripts within a cell under specific conditions. Transcriptomic sequencing can provide information on a wide variety of RNA molecules (e.g., mRNA, tRNA, rRNA, and noncoding RNA) which are transcribed by specific cells under specific conditions. Stress acts differentially on the key cells in the meristem (stem cell, rapidly dividing cell) and on the cells with secondary metabolism. So, the transcriptomic analysis is varied in the different cell types or stages, and a precise sampling strategy is the basis for solving biological problems. Transcriptomics can reflect the changes in gene expression in different cell types. Transcriptomics is considered an effective technique for studying differential gene expression, discovering new genes, developing simple sequence repeats (SSR) markers, and for obtaining tissue- and species-specific transcriptional information [27,28]. The plant stress response is multifaceted and involves a complex interplay between signal transduction pathways, plant–pathogen dynamics, pre- and post-translational regulation, secondary metabolite biosynthesis, nitrogen (N) metabolism, and the activity of transcription factors (TFs), kinases, and transporters (Figure 3). In eukaryotes, post-transcriptional regulation is often carried out by microRNAs (miRNAs), which are non-coding, single-stranded, small RNAs ranging between 19 and 25 nucleotides (nt) in length [29,30]. miRNA-Seq empowers studies of functional genomics and transcriptional regulatory mechanisms. Recently, research has revealed that miRNAs regulate the plant stress response by targeting the genes involved in signal transduction, plant–pathogen interactions, and TFs, among other biological processes (Figure 3).

### 2.1. Transcriptomics of Sugarcane Response to Biotic Stress

#### 2.1.1. Sugarcane Diseases

Disentangling the molecular mechanism underlying sugarcane disease resistance is of great significance for producing disease-resistant varieties using molecular breeding techniques, and transcriptomic analyses can empower such efforts. For example, 2015 differentially expressed genes (DEGs) have been detected in smut-infected sugarcane, a disease caused by the fungal pathogen *Sporisorium scitamineum* [31]. These DEGs encode serine/threonine kinases, calcium-sensing proteins, mitogen-activated proteins (MAPs), and nucleotide-binding site leucine-rich repeat proteins (NBS-LRRs). Smut infestation also influences several phytohormone-responsive signal transduction pathways, including the abscisic acid (ABA)-, salicylic acid (SA)-, ethylene (ET)-, and auxin (Aux)-responsive pathways. Sugarcane resistance to smut infection appears to be influenced by several biological processes, including the immune response, protein metabolism, cell wall formation, and polyamine synthesis [32,33,34,35,36]. Wu et al. [37,38] identified a dynamic gene co-expression network and the candidate genes that were associated with sugarcane smut resistance by using a weighted gene co-expression network analysis (WGCNA) coupled with a bulked segregant RNA-seq (BSR-Seq). miRNA-seq analyses of smut-infected sugarcane have revealed that an array of metabolism- and signaling-associated genes are differentially regulated by miRNAs. Among them, the targets of *miR5671*, *miR6478*, *miR5783*, *miR5054*, and *miR5221* play an essential role in phytohormone signal transduction, plant–pathogen interactions, and mitogen-activated protein kinase (MAPK) activity. Recently, 309 target genes were found to correspond to 337 degradation sites, 112 novel miRNAs, and 97 known miRNAs, implying that miRNAs directly cleave to multiple sites within the target genes [39]. Further analyses have revealed that the targeted genes participated in glycerol metabolism, energy production and transformation, post-translational modification, translation, defense, inorganic ion transport and metabolism, and signal transduction. In addition, many miRNA-mediated resistance-related target genes have been found to be enriched in sugarcane, suggesting that miRNA-mediated genetic regulation may be an important aspect of the sugarcane response to the challenge from *S. scitamineum* [40].

The globally distributed sugarcane red stripe disease is caused by the bacterial pathogen *Acidovorax avenae* subsp. *Avenae* (*Aaa*). However, the molecular mechanism underlying sugarcane resistance to this pathogen is poorly understood. Transcriptomic analyses have identified 467 DEGs in drought-stressed and *Aaa*-infected sugarcane. A Gene Ontology (GO) and Kyoto Encyclopedia of Genes and Genomes (KEGG) analyses of these DEGs revealed that they were involved in amino acid metabolism, carbohydrate metabolism, protein translation, transcription, secondary metabolite biosynthesis, and the stress response. *Aaa* infection has been found to promote gene expression related to ET- and jasmonic acid (JA)-responsive pattern recognition receptor (*PRR*)-biosynthesis, cell wall fortification, *NBS-LRRs*, oxidative burst, and pathogenesis [41]. A recent transcriptomic analysis was conducted on both *Aaa*-resistant (‘ROC22′) and -susceptible (‘MY11-610) sugarcane varieties. Seventy-two hours post inoculation, 15,782 DEGs were detected in the susceptible variety and 18,689 DEGs were detected in the resistant variety. These DEGs were primarily related to plant–pathogen interactions, phenylpropionic acid biosynthesis, carbon metabolism, phytohormone signal transduction, and photosynthesis [23].

Sugarcane pokkah boeng disease is caused by the fungus *Fusarium verticillioides* and, like red stripe disease, the molecular mechanism underlying sugarcane resistance to this pathogen is not well understood. Transcriptomic analyses of the resistant sugarcane variety ‘YT94/128′ and the susceptible variety ‘GT37′ before and after inoculation with *F. verticillioides* revealed 9092 DEGs in the ‘YT 94/128′ and 9829 DEGs in the ‘GT37′. These DEGs were primarily related to the extracellular environment, phenylpropionic acid metabolism, and catalytic enzyme activity, including phosphotransferase, endopeptidase, protein kinase, aldehyde dehydrogenase, oxidoreductase, cellular protease, hydrolase, and peptidase. Further KEGG analysis revealed that these DEGs were related to phenylpropionic acid biosynthesis and metabolism, wax biosynthesis, N metabolism, secondary metabolite biosynthesis, and plant–pathogen interaction [24].

Sugarcane leaf scald disease is caused by the bacterium *Xanthomonas albineans*. Transcriptomic analyses of the resistant sugarcane variety ‘LCP85-384′ and the susceptible variety ‘ROC20′ detected 105,783 *X. albineans*-responsive DEGs primarily involved in endoplasmic reticulum protein processing, spliceosome, glutathione metabolism, phytohormone signal transduction, and plant–pathogen interaction. Furthermore, both the Aux and ET signal transduction pathways were found to be central to the sugarcane response to *X. albineans* infection [25]. Sorghum mosaic virus infection also imposes yield reductions on sugarcane by regulating the expression of some key genes [42,43].

#### 2.1.2. Plant Growth-Promoting Rhizobacteria

The plant growth-promoting rhizobacteria (PGPR) *Burkholderia anthina* ‘MYSP113′ is a sugarcane root-associated diazotroph. Transcriptomic analyses of MYSP113-inoculated sugarcane roots have identified several DEGs related to nitrogen metabolism and phytohormone signal transduction, among other biological processes [44].

### 2.2. Transcriptomics of Sugarcane Response to Abiotic Stress

#### 2.2.1. Nutrient Deficiency

Due to its extensive biomass production, sugarcane has a high requirement for potassium (K). In China, low soil K has resulted in decreased sugarcane yields. Because of this, the leading goal of southern Chinese breeders is to improve the tolerance of sugarcane to low soil K. However, the molecular mechanism underlying sugarcane tolerance to low soil K is not well studied. Transcriptomic analyses of K-deficient sugarcane have identified 4153 genes, with the number of K-deficient responsive genes at 72 h nearly double the number at 8 h and 24 h after a low-K treatment. The primary genes were transcription factors, transporters, kinases, oxidative stress-related genes, and genes in Ca+ and ethylene signaling pathways [45], suggesting that they may play important roles in sugarcane responses to K deficiency.

#### 2.2.2. Aluminum Toxicity

High concentrations of aluminum (Al^3+^) ions can be quite toxic to sugarcane, particularly in acidic soil environments. A transcriptomic analysis of the Al-treated roots of Al-sensitive (‘RB855453’) and Al-tolerant (‘CTC-2’) cultivars detected 1307 DEGs in ‘RB855453’ and 4858 DEGs in ‘CTC-2’, relative to the control plants. These DEGs were grouped into 34 categories of functions, and most were downregulated in ‘RB855453’ and upregulated in ‘CTC-2’. A functional analysis indicated that resistance to Al toxicity in sugarcane may be due to the presence of efficient detoxification mechanisms, the activation of redox enzymes, and lateral root formation [46]. In another study, both Al-tolerant and Al-sensitive sugarcane varieties were screened for miRNAs after an Al treatment. A total of 394 differentially expressed miRNAs (DEMiRs) were detected across both genotypes, among which 87 were common to both genotypes, 116 were specific to the sensitive genotype, and 104 were specific to the tolerant genotype. The identified *DEMiRs* were found to participate in root development, lateral root formation, and signaling [47].

#### 2.2.3. Drought Stress

Water scarcity is one of the most severe limitations for sugarcane production globally. Recently, transcriptomic analyses were carried out on ‘GT21′ sugarcane subjected to mild, moderate, and severe drought at the elongation stage. In all, 1501 DEGs were identified across all the treatments. Of these, 901 DEGs were classified into 36 GO categories and 325 DEGs were classified into 101 pathway categories, including ribosomes, carbon metabolism, and secondary metabolite biosynthesis [48].

A recent RNA-Seq analysis found that drought-sensitive sugarcane varieties (‘RB855453’) respond differently to a water deficit than drought-tolerant varieties (‘SP81-3250’). Specifically, the drought-sensitive genotype exhibited the highest differential gene expression sooner (30 days) than the drought-tolerant genotype (90 days) after drought treatment. These results imply that susceptible genotypes require more rapid response mechanisms to effectively react to water deficit conditions [49]. In another experiment, the drought-sensitive variety ‘Co 8021’ and the drought-tolerant variety ‘Co 06022’ were subjected to mild, moderate, and severe drought and re-watering, and the leaves were subjected to RNA-Seq. In the drought-tolerant variety, 2970 unigenes were identified after 2 days of drought stress, 2109 unigenes identified after 6 days of drought stress, 2307 unigenes were identified after 10 days of drought stress, and 1334 unigenes were identified 10 days after re-watering. Fewer unigenes were identified in the drought-susceptible variety, with 2025 unigenes identified after 2 days of drought stress and 1552 unigenes identified after 6 days of drought stress. However, upon re-watering, more unigenes were identified in the drought-susceptible variety, implying that drought-sensitive sugarcane may need to upregulate a greater number of genes and processes in order to recover from drought stress. The drought-tolerant variety was found to contain nearly twice as many DEGs between the different stages as compared to the drought-susceptible variety. Across both varieties, several genes which underlie important metabolic functions were found to be responsive to a water deficit, as were many novel genes with unknown functions [50].

A similar experiment was performed using the drought-tolerant variety ‘KPS01-12’ and the drought-sensitive variety ‘UT12’, which were subjected to mild and moderate drought stress. Compared to the drought-sensitive variety, more genes related to antioxidant secondary metabolite biosynthesis, oxidative and osmotic stress response, and water retention were upregulated in the drought-tolerant variety. In addition, more genes related to the Calvin cycle, carbon fixation, and photosynthesis were downregulated in the drought-sensitive variety compared to the drought-tolerant variety. Taken together, these results suggest that the drought-tolerant sugarcane varieties have a more effective drought response mechanism than the drought-sensitive varieties [51].

Wild species and the relatives of sugarcane are important sources of biotic and abiotic stress resistance genes. Despite this, the genes associated with drought tolerance are poorly understood in wild sugarcane species. To address this knowledge gap, a recent study subjected *Saccharum narenga* to 22 days of drought stress in order to study the drought-associated transcriptomic changes. A total of 3389 DEGs were detected in the *S. narenga* leaves, many of which were involved in the response to blue light, metabolism, and phytohormone signal transduction. Interestingly, the different subfamilies of ribosomal proteins and aquaporins exhibited differential regulation, with DIVARICATA and heat stress-associated TFs being the first responders. In addition, several miRNAs were predicted to be involved in the sugarcane response to a water deficit [52].

The polyploid *S. spontaneum* is the ancestor of modern sugarcane. A recent study subjected *S. spontaneum* ‘GXS87-16’ to mild, moderate, and severe drought, and re-watering at the elongation stage. RNA-Seq identified a total of 1569 DEGs, with the majority being induced by the water deficit. GO and KEGG analyses assigned these DEGs to 47 GO categories and 93 metabolic pathways associated with phytohormone signal transduction, plant–pathogen interaction, RNA transport, and mRNA surveillance [53]. In another study, three-month-old drought-tolerant (‘RB867515’) and drought-sensitive (‘RB855536’) sugarcane plants were subjected to 2, 4, 6, or 8 days of drought stress in order to identify the miRNAs involved in regulating the drought stress response. In total, 18 miRNA families were identified, seven of which were differentially expressed during a drought. Six miRNAs were found to be differentially expressed after 2 days of drought stress and five were differentially expressed after 4 days of drought stress. Six precursors, as well as the targets, of the DEMiRs were predicted [54]. Another miRNA analysis was also conducted on the drought-resistant variety ‘ROC22’ treated with PEG to simulate a drought. A total of 57 miRNA families were identified, among which 34 were unknown and 23 were known. In addition, 438 target genes were predicted to be the targets of 44 miRNA families. Eleven miRNA families were differentially expressed in response to PEG treatment, with many of the predicted targets associated with the plant stress response, including *MYB*, *BCP*, *CPI*, *NCBP*, *SPBP*, *LSG*, and *AGO1-like* [55].

#### 2.2.4. Extreme Temperatures

Because sugarcane is produced in tropical and sub-tropical climates, this crop is considered particularly vulnerable to the increasing temperatures associated with climate change. To identify the heat-responsive genes, a transcriptomic analysis was performed on the sugarcane variety ‘Co 99004’, subjected to high temperatures (47 °C), resulting in the detection of 1137 genes which were upregulated. Specifically, *phytepsin*, *stress protein DDR-48*, and *ferredoxin-dependent glutamate synthase* exhibited a three-fold higher expression in the heat-stressed plants, relative to the control plants [56].

### 2.3. Transcriptomics of Sugarcane Response to Exogenous Phytohormone Application

A high sucrose content is the primary goal of sugarcane breeding. Unfortunately, recent decades have seen little success in improving the sucrose content of sugarcane through conventional breeding techniques. By mining the key genes regulating sucrose accumulation, molecular breeding techniques are poised to break through the limitations of conventional breeding.

Ethylene treatment is a proven way to increase sucrose in sugarcane. Transcriptomic analyses of low-, medium-, and high-sugar varieties of sugarcane identified approximately 25,000 ethylene-responsive DEGs. However, the genotype exhibited a more significant effect on the number of differential expressed genes than ethylene exposure. Notably, the genes involved in sucrose and starch metabolism were more sensitive to ethylene exposure in the low-sugar varieties. The phytohormone ET induces alterations in gene expression patterns related to epigenetic modification, phytohormone metabolism, stress-related TFs, and carbohydrate metabolism. Furthermore, ET induces the expression of genes related to *ATPase*, cell wall-binding invertase, cytoplasmic acid, and ET-responsive TFs more strongly in the low-sugar varieties than in the high-sugar varieties. These results suggest that ethylene treatment enhances the sink strength of the low-sugar varieties of sugarcane, thus enhancing sucrose accumulation [57].

The phytohormone gibberellic acid (GA_3_) has been shown to increase sucrose accumulation, sink strength, and internode length in sugarcane. A transcriptomic analysis of the internodes of the high-sugar variety ‘CoLk94184’ treated with exogenous GA_3_ identified a total of 201,184 transcripts. Specifically, 1516 differentially expressed transcripts (DETs) were identified in the bottom internodes and 1589 DETs were identified in the top internodes. These DETs were grouped into 153 functional categories based on a KEGG analysis. Of these, the DETs involved in starch and sucrose metabolism exhibited a 5.0 fold change (FC) in the top internodes and a 3.0 FC in the bottom internodes [58]. The transcripts identified in this study could be used to provide insights into the factors/genes affecting sucrose accumulation in sugarcane.

The formation of adventitious roots (ARs) on sugarcane microshoots is enhanced by treatment with the Aux-type phytohormones 1-naphthalene acetic acid (NAA) and indole-3-butyric acid (IBA). In a recent study, basal microshoot tissues (5 mm) were subjected to NAA and IBA treatment, with water as the control. Transcriptomic analyses identified 1737 and 1268 DEGs between the experimental and control tissues on the 3rd and 7th day of treatment, respectively. GO and KEGG analyses indicated that these DEGs were related to cell wall modification, flavonoid and phenylpropanoid biosynthesis, cell cycle, and phytohormone signaling. Furthermore, several TFs appear to be regulated by Aux-mediated AR formation [59]. It appears that the phytohormones played a key role in the transition of the shoot cells to root meristem and then to AR through cell wall modification and synthesis, cell proliferation, root meristem identity preservation, and cell growth.

## 3. Role of Transcriptomics in Studies of Environmentally Stressed Sugarcane

Molecular analyses have identified a variety of genes (mainly involved in the biosynthesis of secondary metabolites, ribosomes, and carbon metabolism) in sugarcane which are responsive to stressful environmental conditions [48,60]. These studies utilized in silico techniques, such as expressed sequenced tags (ESTs) and probe hybridization arrays, to study the genes in sugarcane and allied crops. For example, the extensive Brazilian sugarcane EST database contains approximately 238,000 ESTs from 26 cDNA libraries covering a diverse array of tissues and plant varieties [61]. However, our current lack of a comprehensive sugarcane genome continues to hinder transcriptomic studies of this important crop [62]. Because of this, the *Sorghum bicolor* reference genome is often substituted in transcriptomic studies of sugarcane, due to the high genetic similarity between the two crops [63,64]. 

Transcriptomic studies of stressed sugarcane plants can reveal the molecular mechanisms underlying stress resistance and provide resources for genetic improvement. Such studies have shown that a variety of TFs in plants, including *zinc-finger*, *AP2/DREBP*, *bZIP*, *MYB*, and *WRKY*, are responsive to environmental stress [65]. Worldwide, a variety of environmental stressors are responsible for decreased sugarcane production [4]. Transcriptomic studies of stress-tolerant and -susceptible sugarcane varieties often make use of the Illumina HiSeq 2500 and HiScanSQ systems [49]. The use of these, and other, systems has revealed that several genes, including those encoding *aquaporins*, *co-enzyme A ligases*, *E3 SUMO-Protein ligase SIZ2*, *MYB* TFs, and *ascorbate peroxidase 3* (*APX3*), are upregulated in the stress-tolerant varieties [49,66,67]. 

The expression of ABA-sensitive genes is upregulated under drought and high temperatures. da Silva et al. [47] reported similar observations in an HT-SuperSAGE study of drought-tolerant and -sensitive sugarcane varieties. They identified 9.831 unitag genes with differential regulation in both drought-sensitive and -tolerant varieties. Many of these genes were related to the pentose phosphate pathway, carbohydrate metabolism, amino acid transport, fatty acid biosynthesis, oxidative detoxification, protein degradation, root growth, and ET stress [68]. In another experiment, wild type (WT) *S. narenga* was subjected to drought stress. The authors identified 3389 upregulated and downregulated DEGs, many of which were related to phytohormone signal transduction, blue light response, and metabolism [52]. 

Several genes have been linked to stress alleviation in sugarcane, including *thioredoxin*-, *S-adenosylmethionine* (*SAM*) *decarboxylase*-, *cytochrome c oxidase*-, and *polyamine oxidase*-encoding genes [48,60]. Genes encoding *delta-12 oleate desaturase*, *S-adenosylmethionine decarboxylase*, and *protein phosphatases* (*PP2C*) have been shown to be upregulated by both drought stress and ABA. Furthermore, the ET-responsive factor *SodERF3* has been found to be upregulated by drought and ABA in sugarcane. Differential gene expression patterns are indicators of complex defense mechanisms which aid in defending cells from drought-induced damage [48,69]. High-throughput microarrays are well suited for DEG analyses, as they allow several genes to be evaluated simultaneously. One recent study identified 15,593 expressed genes in sugarcane, with 1501 of these exhibiting differential expression under stressful conditions [51]. Such results are often subjected to functional annotation to determine the biological processes and pathways associated with DEGs [45].

A DEG survey was recently conducted in cold-stressed *S. spontaneum*, resulting in the identification of 5840 cold-responsive genes, including 3302 downregulated genes and 2538 upregulated genes [70]. Other studies have sought to evaluate transcriptomic changes in sugarcane exposed to low-K and low-N conditions [45,71] and to cold stress [72]. Studies have additionally sought to identify the proteins associated with cold tolerance by studying the expression of cold-responsive genes. For example, one EST encoding a putative *xanthine dehydrogenase* (*XDH*) [73] was found to be upregulated in sugarcane exposed to freezing temperatures [74,75]. In another study, 165 DEGs were associated with 3575 ESTs in stress-tolerant sugarcane plants [76]. The ESTs were clustered according to the species from which they were derived: two small groups representing *Saccharum arundinaceum* and *S. officinarum*, and one large group representing *Saccharum* spp. hybrids.

Transcriptomic studies of sugarcane have utilized advanced molecular techniques, such as microscopy, qPCR, RNA-Seq, Solexa, Illumina, Roche, and 454 sequencing, and cDNA microarrays [19,77]. Analyses of the agronomically relevant structural and functional changes associated with gene expression are crucial for crop improvement. Transcriptomics can be used to corroborate and explain gene expression patterns [78]. Normal sugarcane growth and development requires dynamic expression changes in the genes related to gas exchange, leaf maturation, leaf abscission, and cellulose and lignin biosynthesis [6,79,80,81]. To aid sugarcane crop improvement, current and future transcriptomic studies should focus on environmental stress avoidance and sucrose accumulation.

## 4. Sugarcane Proteomic Analysis

**Figure 4:**
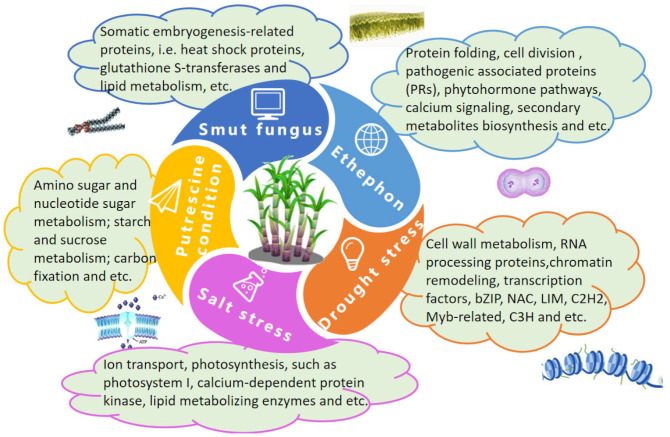
The sugarcane stress response involves multiple biological processes, signal transduction pathways, and proteins.

The proteome includes all the proteins expressed in a particular cell or tissue. Proteomics is a feature of post-genomic science [82] and proteomic techniques can explain how genes are regulated, identify key factors or life processes, and quantify protein expression. In sugarcane, proteomics can aid in identifying and characterizing the proteins associated with the stress response. Such studies have identified several biological processes associated with the stress response in sugarcane, including TFs, chromatin remodeling, RNA processing, cell wall metabolism, photosynthesis, and ion transport (Figure 4).

### 4.1. Proteomic Analyses of Sugarcane Subjected to Biotic and Abiotic Stressors

#### 4.1.1. Sugarcane Diseases

One recent proteomic study of the meristematic tissue from a smut-susceptible sugarcane variety infected with *S. scitamineum* at the whip emergence stage identified 53 differentially expressed proteins (DEPs). These DEPs were related to cell division, protein folding, stress, metabolism, and defense. In addition, the putative effector chorismate mutase was identified in *S. scitamineum*. Interestingly, both the expression and activity of phenylalanine ammonia-lyase were increased in the smut-infected meristematic tissue [18]. In another proteomic analysis of *S. scitamineum*-infected sugarcane, 341 DEPs were identified in the smut-susceptible variety ‘ROC22’ and 273 DEPs were identified in the smut-resistant variety ‘Yacheng05-179’. However, the proteomic results were poorly correlated with the transcriptomic results (0.2466 and 0.1502, respectively), suggesting that post-translational events may be important mediators of the relationship between sugarcane and *S. scitamineum*. Many of the DEPS were linked with smut resistance, including heat shock proteins, lectins, pathogenic associated protein 1 (PR1), peroxidase, β-1,3-glucanase, and endo-1,4-β-xylanase. In addition, the ET and GA_3_ pathways, phenylpropionic acid metabolism, and several pathogen-related proteins (PR5, PR2, PR1, and PR14) were upregulated in the smut-resistant variety ‘Yacheng05-179′. Interestingly, the ABA, reactive oxygen species, nitric oxide, and calcium signaling pathways were downregulated by a *S. scitamineum* infection in ‘Yacheng05-179’, suggesting that these pathways may be less important for defense [83].

In a more recent study, 2D-PAGE was used to survey DEPs in smut-susceptible (‘NCo310’) and smut-resistant (‘F134’) sugarcane varieties before and after *S. scitamineum* inoculation. A total of 30 DEPs were detected, including 16 from ‘F134’ and 14 from ‘NCo310’. Four DEPs were upregulated and nine downregulated in ‘F134’, while nine DEPs were upregulated and three downregulated in ‘NCo310′. These DEPs were associated with the plasma membrane, nucleus, chloroplast, protein renaturation, stress response, photorespiration, metabolism, defense, and DNA binding [84], which was similar to the other proteomic report by Que et al. [85].

The proteomes of the red stripe-resistant variety ‘LCP85-384’ and -susceptible variety ‘ROC20’ were evaluated before and after *X. albineans* inoculation. In total, 4295 DEPs related to 1099 GO classifications were detected. Among these, 285 DEPs were detected in both ‘LCP85-384’ and ‘ROC20’, 172 DEPs were detected in the resistant variety ‘LCP 85-384’, and 192 DEPs were detected in the susceptible variety ‘ROC20’. Many of the DEPs significantly upregulated in ‘LCP85-384’ were found to be related to phenylpropionic acid biosynthesis, secondary metabolite biosynthesis, and other metabolic pathways [86].

#### 4.1.2. Drought and Salt Stress

A recent proteomic analysis of sugarcane stems subjected to drought stress identified 5381 protein groups and 1204 drought-responsive DEPs. Among these, 586 DEPs were upregulated and 618 were downregulated. In addition, 115 specific proteins were identified, including 41 from the experimental plants and 74 from the control plants, the majority of which were associated with cell wall metabolism. Thirty-seven drought-responsive TFs were also detected, including Aux-responsive, heat shock, C3H, Myb-related, C2H2, LIM, NAC, and bZIP. These results suggest that an imbalance between protein degradation and protein synthesis was induced by the water deficit [87].

Sugarcane is particularly vulnerable to salt stress and, at present, no sugarcane variety exhibits high salt tolerance. One study surveyed the proteomes of micropropagated shoots of salt-stressed ‘CB38-22’ and ‘RB855536’ sugarcane. Overall, ‘CB38-22’ was found to be less salt tolerant than ‘RB855536’. Compared to ‘CB38-22’, the proteins related to photosynthesis, non-enzymatic antioxidants, and ion transport were upregulated in ‘RB855536’. In addition, several proteins were more abundant in the salt-stressed ‘RB855536’, including phospholipase D, glyceraldehyde-3-phosphate dehydrogenase, calcium-dependent protein kinase, and photosystem I [88].

Another proteomics analysis was carried out on two genotypes with contrasting tolerance to saline soil. Compared to the sensitive genotype, the tolerant genotype showed a 3-fold increase in GDSL-motif lipases and lipid metabolizing enzymes related to the abiotic stress defense. In addition, the abundance of lipoxygenase and Type III chlorophyll a/b-binding proteins were increased 2-fold in the tolerant genotype relative to the susceptible genotype. Furthermore, the key C4-photosynthesis enzyme phosphoenolpyruvate carboxylase, and other chloroplast enzymes, was downregulated in the sensitive genotype. Interestingly, neither variety experienced sodium (Na) toxicity under high salt conditions, suggesting that these proteins may improve osmotic adjustment mechanisms [89].

#### 4.1.3. Ethylene Treatment

Recent decades have seen little improvement in sucrose accumulation in sugarcane, and ethylene treatment can regulate sucrose metabolism in sugarcane. A recent proteomic study identified 2983 proteins in low- and high-sugar sugarcane varieties treated with ethylene, including 139 DEPs, many of which were associated with carbohydrate metabolism. Among these, 25 DEPs were found to regulate sucrose accumulation, including the proteins involved in carbon fixation; fructokinase, α-glucan phosphorylase, and β-D-glucosidase, related to starch and sucrose metabolism; and UDP-glucose 6-dehydrogenase, related to amino sugar and nucleotide sugar metabolism [90].

#### 4.1.4. Putrescine Treatment

A proteomic analysis of putrescine-treated and untreated sugarcane identified several somatic embryogenesis-related DEPs which are related to stress resistance, including 14-3-3 proteins, late embryogenesis abundant proteins, glutathione S-transferases, heat shock proteins, peroxidases, and arabinogalactan proteins [91].

## 5. Role of Proteomics in Studies of Environmentally Stressed Sugarcane

Both proteomic and transcriptomic studies help to illuminate complicated biological mechanisms [92,93]. While the genome is “static”, the proteome responds dynamically to changing internal and external conditions through post-translational modifications, such as acetylation, methylation, phosphorylation, and glycosylation, as well as other mechanisms [93]. Thus, to comprehensively characterize biological phenomena, protein expression must be quantified, proteins must be functionally evaluated, and post-translational derivates must be identified. Several protein quantification and isolation techniques, such as matrix-assisted laser desorption/ionization-time of flight-mass spectrometry–two-dimensional electrophoresis, have been used to study differential protein expression in sugarcane [93]. iTRAQ is also a powerful quantitative proteomic technique [94]. In addition, many sugarcane studies have used both gel-free and gel-based methods.

Environmental stress directly and indirectly alters the metabolic, morphological, and physiological state of sugarcane [93]. Abiotic stress is considered multidimensional in that it interacts with multiple cellular and metabolic processes, resulting in a loss of productivity [95]. The balance between protein synthesis and degradation has a significant effect on stress resilience, as shown in several proteomics studies of stress-tolerant sugarcane varieties. Liquid chromatography-electrospray ionization-mass spectrometry (LC-ESI-IT-MS/MS/MS) coupled with 2-DE was utilized to detect proteins related to photosynthesis and antioxidation [96]. In another study utilizing liquid chromatography-mass spectrometry (LC-MS/MS) coupled with 2-DE, several drought-responsive proteins were identified in both the tolerant and sensitive sugarcane varieties [95]. In another study utilizing MS/2-DE methods, several proteins were found to be differentially expressed in salt-stressed sugarcane, such as heat shock proteins, glyceraldehyde 3-phosphate dehydrogenase, germin-like protein, and fructose 1,6-bisphosphate aldolase [97]. Heat shock proteins (HSPs) are well known to mitigate environmental stress in plants [98]. 

While proteomic techniques have been used to study the tissue- and growth stage-specific protein profiles of sugarcane, a more comprehensive evaluation of the sugarcane proteome over the entire developmental process is needed. In comparison to mature tissue, young sugarcane leaves and stems have been found to exhibit high rates of lipid metabolism and to be enriched in 277 cell wall proteins (CWPs). A more comprehensive proteomic evaluation of sugarcane across all the developmental stages will aid in the development of genetically modified sugarcane varieties through the use of gene transformation and molecular breeding techniques [99,100].

## 6. Conclusions and Perspectives

**Figure 5:**
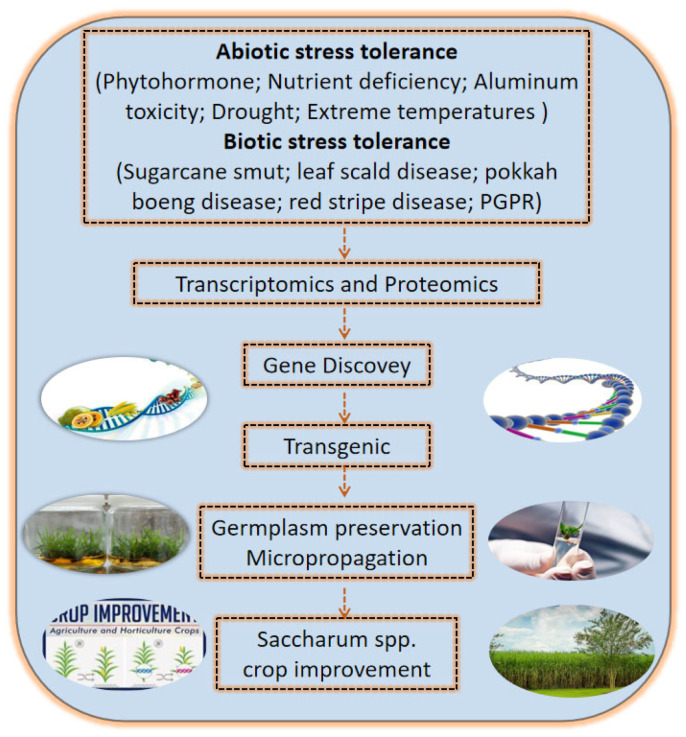
Different approaches for the improvement of sugarcane.

Sugarcane productivity is both indirectly and directly affected by climatic conditions. It is therefore a central goal of both agro-scientists and policymakers to alleviate the harmful effects of stressful environmental conditions on sugarcane and to improve sugarcane productivity. Such an undertaking will require a multidisciplinary approach to consistently develop new sugarcane varieties through traditional and molecular breeding techniques (Figure 5). Advanced transcriptomic and proteomic techniques will be necessary to understand how various stresses affect sugarcane’s molecular mechanism and production and to fill in the existing research gaps. For breeders, uncovering the physiological mechanism and trying to link the molecular mechanism with the physiological one is essential for crop improvement.

## Data Availability

No new data were created.
